# Sphingomyelin 14:0 ‐ a driver of neuroinflammation

**DOI:** 10.1002/alz.089428

**Published:** 2025-01-03

**Authors:** Roisin M. McManus

**Affiliations:** ^1^ German Center for Neurodegenerative Diseases (DZNE), Bonn, NRW, Germany; Institute of Innate Immunity, Bonn, NRW Germany

## Abstract

**Background:**

Western‐diet (WD) can induce sterile inflammation and epigenetic reprogramming of myeloid cells, affecting their immune response (Christ et al., 2018). However, the molecular signaling mediating these changes was unknown. We recently identified that WD increases circulating sphingomyelin 14:0 (S14), which was associated with peripheral inflammation and atherosclerosis. As atherosclerosis, inflammation and WD are risk factors for dementia and Alzheimer’s disease (AD), our goal was to determine whether S14 was the precise signaling factor driving neuroinflammation leading to AD.

**Method:**

Wild‐type and *Apoe* ^‐/‐^ mice (mouse model of atherosclerosis) were fed a high‐fat diet mimicking WD for 8 weeks, before samples were obtained for lipidomics. Microglia were isolated *ex vivo* for either RNA sequencing or to determine their metabolic profile. In a separate series of experiments, microglia were prepared from wild‐type mice and treated with various TLR inhibitors to assess whether these cells can recognize S14, and the exact molecular pathways induced by the lipid. Wild‐type microglia were prepared for RNA sequencing, to determine the genes and networks induced by S14, which were compared with the microglia enriched *ex vivo*. Pathways were confirmed at a protein and functional level.

**Result:**

Lipidomic analysis confirmed that WD increased S14 in the circulation of wild‐type and *Apoe* ^‐/‐^ mice. Patients with obesity also had greater levels of S14, which was associated with heart disease. We found that S14 triggers an extensive increase in pro‐inflammatory genes and pathways in microglia. We confirmed that S14 utilizes TLR4 to signal *in vitro*, inducing NFkB activation and ultimately the release of TNFα from microglial cells. S14 also affected microglial metabolic function and reduced phagocytic capacity *ex vivo* and *in vitro*.

**Conclusion:**

We found that S14 is a lipid significantly increased in both human and murine models of obesity or heart disease. S14 is a novel TLR4 agonist that can activate microglia and trigger extensive inflammation. Critically, S14 affects microglial function both *ex vivo* and *in vitro*. Together, our data shows that S14 is an endogenous immune stimulus, capable of triggering various innate immune responses and microglial changes that are associated with neuroinflammation and the development of dementia.

